# Establishment and characterization of cytochrome P450 1A1 CRISPR/Cas9 Knockout Bovine Foetal Hepatocyte Cell Line (BFH12)

**DOI:** 10.1007/s10565-024-09856-7

**Published:** 2024-03-26

**Authors:** Silvia Iori, Caterina D’Onofrio, Nihay Laham-Karam, Isidore Mushimiyimana, Lorena Lucatello, Rosa Maria Lopparelli, Maria Elena Gelain, Francesca Capolongo, Marianna Pauletto, Mauro Dacasto, Mery Giantin

**Affiliations:** 1 Department of Comparative Biomedicine and Food Science, University of Padua, Viale Dell’Università 16, Legnaro, 35020 Padua, Italy; 2 University of Eastern Finland, A.I. Virtanen Institute for Molecular Sciences, Neulaniementie 2, 70211 Kuopio, Finland

**Keywords:** CRISPR/Cas9, CYP1A1, Knockout, Bovine, Liver cells, Transcriptome

## Abstract

The cytochrome P450 1A (CYP1A) subfamily of xenobiotic metabolizing enzymes (XMEs) consists of two different isoforms, namely *CYP1A1* and *CYP1A2*, which are highly conserved among species. These two isoenzymes are involved in the biotransformation of many endogenous compounds as well as in the bioactivation of several xenobiotics into carcinogenic derivatives, thereby increasing the risk of tumour development. Cattle (*Bos taurus*) are one of the most important food-producing animal species, being a significant source of nutrition worldwide. Despite daily exposure to xenobiotics, data on the contribution of *CYP1A* to bovine hepatic metabolism are still scarce. The CRISPR/Cas9-mediated knockout (KO) is a useful method for generating in vivo and in vitro models for studying xenobiotic biotransformations. In this study, we applied the ribonucleoprotein (RNP)-complex approach to successfully obtain the KO of *CYP1A1* in a bovine foetal hepatocyte cell line (BFH12). After clonal expansion and selection, *CYP1A1* excision was confirmed at the DNA, mRNA and protein level. Therefore, RNA-seq analysis revealed significant transcriptomic changes associated with cell cycle regulation, proliferation, and detoxification processes as well as on iron, lipid and mitochondrial homeostasis. Altogether, this study successfully generates a new bovine *CYP1A1* KO in vitro model, representing a valuable resource for xenobiotic metabolism studies in this important farm animal species.

**Graphical Abstract:**

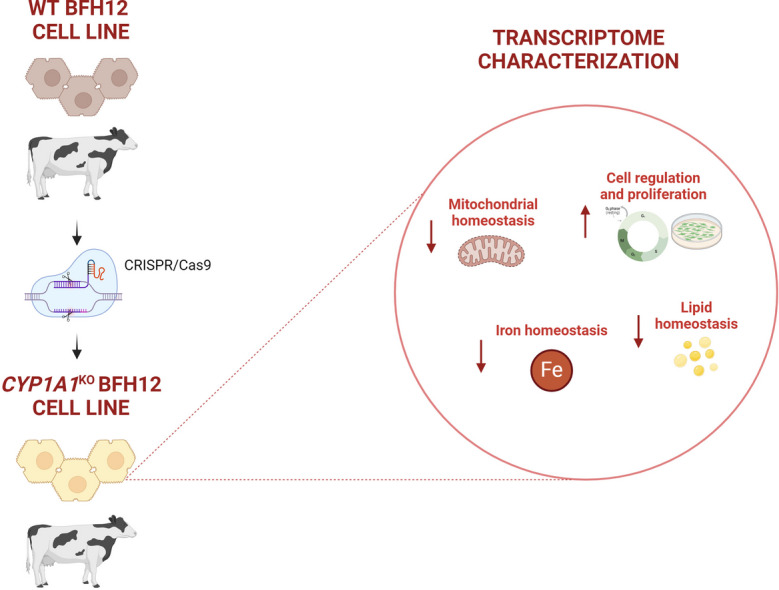

**Supplementary Information:**

The online version contains supplementary material available at 10.1007/s10565-024-09856-7.

## Introduction

It’s a fact that the cytochrome P450 (CYP) gene superfamily of microsomal haemoproteins is the most important group of xenobiotic metabolizing enzymes (XMEs) involved in oxidative phase I biotransformations, that result in either bioactivation or detoxification reactions of a broad spectrum of endogenous and exogenous substrates. Among the main members of this enzyme superfamily the CYP1A gene family is included. It consists of two different isoforms, i.e. *CYP1A1* and *CYP1A2*, which are highly conserved among species. In humans, the *CYP1A2* isoform is constitutively and highly expressed in the liver; conversely, *CYP1A1* is considered an extrahepatic isoform, mostly expressed in the intestine, lungs, brains, placenta and kidney, but inducible in the liver (Nebert et al. 2004). These two XMEs are involved in the biotransformation of many endogenous compounds such as retinol, linoleic acid, melatonin, progesterone and estradiol (Lu et al. 2020) as well as of about 10% of marketed drugs (Zanger and Schwab 2013). Furthermore, CYP1As play a crucial role in the bioactivation of a number of chemicals into carcinogenic and/or reactive derivatives, thereby increasing the risk of tumour development. Among these xenobiotics, we can count environmental pollutants like polycyclic aromatic hydrocarbons, and mycotoxins (Goedtke et al. 2021; Mary et al. 2015; Zhang et al. 2022).

The expression of *CYP1A* is mainly regulated by the aryl hydrocarbon receptor (AHR) that in turn, once activated, may lead to the coordinated transcription of both isoforms (Gargaro et al. 2021). In this respect, some AHR ligands such as benzo[a]pyrene (a persistent organic pollutant; POP), the mycotoxin aflatoxin B1 (AFB1), and drugs like omeprazole and lansoprazole are considered strong *CYP1A* inducers (Arenas-Huertero et al. 2019; Goedtke et al. 2021; Zhang et al. 2022)*.* Worth to mention, *CYP1A* induction is also likely to occur following the activation of another transcription factor, i.e. the constitutive androstane receptor (CAR); indeed, typical CAR ligands such as carbamazepine, phenobarbital, and phenytoin may determine *CYP1A* transactivation (Yoshinari et al. 2010). All the aforementioned compounds, but theoretically others too, may contribute to the variability in the expression and function of both CYP1A1 and CYP1A2 apoproteins in humans.

Cattle (*Bos taurus*) are considered a major food-producing animal worldwide, that during their life are exposed to xenobiotics such as drugs, pesticides, growth hormones, feed additives and environmental contaminants. These compounds as well as their metabolites may end up in cattle-derived food products, like milk, cheese and edible tissues, thus becoming harmful not only for the animal itself but also for consumers.

Consequently, cattle xenobiotic metabolism studies are crucial in the risk assessment associated to the consumption of bovine food products. Despite this major concern, the molecular mechanisms involved in cattle CYP1A expression and regulation have been poorly investigated so far, and most of the available information refers to the contribution of CYP1A to xenobiotic metabolism.

A previous phylogenetic study grouped bovine *CYP1A1* and *CYP1A2* genes with the corresponding human orthologous sequences (Zancanella et al. 2010), confirming the high conservation of these isoforms among mammalian species. In line with this assumption, they were both detected in the liver of different cattle breeds (Giantin et al. 2008) at the mRNA and protein levels; moreover, a further study conducted on bovine liver slices not only confirmed the constitutive expression of both isoforms, but also the relatively higher expression of *CYP1A2* (Maté et al. 2019) compared to that of *CYP1A1*. As to CYP1A-dependent catalytic activity, an efficient *O*-deethylation of ethoxyresorufin (EROD) and methoxyresorufin (a CYP1A1- and CYP1A2-mediated biotransformation, respectively) were observed in cattle (Pegolo et al. 2010; Cantiello et al. 2022). Additionally, the bovine hepatic *CYP1A* is induced by the prototypical CYP1A inducer β-naphthoflavone (βNF) (Maté et al. 2019).

At present, very few hepatic in vitro models are available to clarify the mechanisms involved in bovine hepatic CYP1A expression, regulation and catalytic activity. Among these ones, the bovine foetal hepatocyte-derived cell line BFH12 (Gleich et al. 2016) may be considered as the most suitable in vitro tool for the execution of molecular studies on cattle CYP1A. Indeed, this cell line shows a hepatocyte-*like* metabolism and a stable expression of several XMEs and drug transporters (Gleich et al. 2019). In particular, *CYP1A* mRNA may be induced by two prototypical CYP1A inducers, i.e. βNF and 3,3',4,4',5-pentachlorobiphenyl (PCB126) (Giantin et al. 2018; Pauletto et al. 2020); moreover, its transcriptional response following the exposure to the hepatotoxic and hepatocarcinogenic AFB1 was recently described (Pauletto et al. 2020; Iori et al. 2022). Even though *CYP1A* pattern of expression is quite different from the *ex vivo *situation (*CYP1A1* is the most expressed *CYP1A* isoform: Giantin, personal data), the BFH12 cell line allows researchers to study not only the bovine CYP1A-dependent xenobiotic metabolism but also the molecular mechanisms involved in CYP1A regulation of gene expression.

The CRISPR/Cas9 system is extensively applied to knockout (KO) a gene of interest and indirectly define its contribution to a given outcome. Several CYP-KO rodent models, including *CYP1A1*^KO^ and *CYP1A2*^KO^, were established for the study of xenobiotic hepatic metabolism and pharmacokinetics (Kapelyukh et al. 2019; Sun et al. 2021; Lu et al. 2023). However, results obtained from laboratory animal models cannot be directly applied to livestock species, due to known species-specific differences in XMEs expression, regulation and function.

With the aim of establish a new in vitro model useful to gain more insights into bovine hepatic CYP1A expression, regulation and enzyme activity, we applied the CRISPR/Cas9 technology in BFH12 cells to excise *CYP1A1* gene. To meet this goal, a ribonucleoprotein (RNP)-complex transfection was performed. Then, the *CYP1A1* deletion was assessed through DNA Sanger Sequencing, and confirmed at the mRNA, protein and catalytic activity level comparing the engineered cell model (*CYP1A1*^KO^) with the control line (*CYP1A1*^CTL^). Finally, the transcriptome of the obtained *CYP1A1*^KO^ cell model was characterized by RNA-sequencing (RNA-seq).

## Materials and methods

### Reagents and chemicals

Complete William’s E Medium, Lipofectamine™ CRISPRMAX™ Cas9 Transfection Reagent, Phire Hot Start II DNA Polymerase, LightCycler 480 PowerUp™ SYBR® Green Master Mix, Qubit RNA Assay Kit, High Capacity cDNA Reverse Transcription Kit, SuperSignal® West Pico chemiluminescence substrate and BCA Assay Kit were from Thermo Fisher Scientific (Waltham, Massachusetts, USA). DNeasy Blood & Tissue kit and RNeasy Mini kit were purchased from Qiagen (Venlo, The Netherlands). DNA, RNA and Protein Purification kit was provided by Macherey–Nagel (Düren, Germany). Rabbit anti-human beta-actin (ACTB, GTX109639), the peroxidase-conjugated goat anti-rabbit IgG and the rabbit anti-human CYP1A1 antibodies were from GeneTex (Irvine, California, USA).

### Bovine foetal hepatocyte cell line

The cell line (BFH12) was provided by Dr. Axel Schoeniger (Institute of Biochemistry, University of Leipzig, Leipzig, Germany). Cells were maintained in 25 cm^2^ flasks and cultured in Williams’ E medium supplemented with 5% heat-inactivated FBS, 1% penicillin/streptomycin, 2 mM L-alanyl-L-glutamine, 100 nM dexamethasone and 0.2 U/mL insulin.

### Generation of BFH12 *CYP1A1*^KO^ cells

The CRISPR/Cas9-mediated *CYP1A1*^KO^ was performed using the Alt-R CRISPR-Cas9 system (IDT; Coralville, Iowa, USA). The guide RNA sequences (gRNAs) were designed using CRISPOR tool (http://crispor.tefor.net/) and ordered as target-specific crisprRNA (Alt-R® CRISPR-Cas9 crRNA). Specifically, to design the gRNAs, various parameters provided by the CRISPOR tool (Supplementary Table 1), including the MIT and CFD specificity scores, the number of off-targets for mismatches and the predicted cleavage efficiency score (Doench score method: Doench et al. 2016; Haeussler et al. 2016) were considered. Table 1 shows the selected crRNAs (i.e., CYP1A1#1 and CYP1A1#2) among the four sequences tested in the preliminary setup. Four days prior to transfection, BFH12 cells were seeded into a 96-well plate (4 × 10^3^ cells/well). On the day of transfection, crRNAs were annealed to the labelled trans-activating crRNA (Alt-R™ CRISPR-Cas9 tracrRNA, ATTO™ 488) and the Cas9 enzyme (Alt-R® S.p. HiFi Cas9 Nuclease V3) to form RNP-complexes, following IDT protocol. These latter ones were then delivered into BFH12 cells by lipofection, using Lipofectamine CRISPRMAX reagents and following the IDT protocol with some modifications. Briefly, 7.5 µL of RNP-complexes were mixed with 1.2 µL of CRISPRMAX reagent and 23.8 µL of Opti-MEM Media. After 30 min, the culture medium was replaced with 50 µL of transfection complex and 100 µL of culture medium without antibiotics. BFH12 cells transfected in the absence of RNP-complex (i.e., transfection reagent only) served as negative control (*CYP1A1*^CTL^). Two days post-transfection cells were collected, assayed for transfection efficiency by flow cytometry analysis (CyFlow Space flow cytometer, Partec-System, Sysmex Europe GmbH, Norderstedt-Amburgo, Germany; Supplementary Fig. 1a) and diluted in Petri dishes for clone selection. The culture medium was changed every 7 days. Once single discrete colonies were formed, they were picked up and propagated for genotyping analysis.

**Table 1:** List of crRNAs used for CRISPR/Cas9 mediated KO of bovine *CYP1A1* gene

Guide ID	Sequence	Target region
CYP1A1#1	TAAGAGCCAGCACTTTATCG	Promoter
CYP1A1#2	CGGTGAGACCATTGCCCGCT	3’ UTR

### DNA extraction and PCR-based confirmation of *CYP1A1* correct deletion

**Fig. 1:**
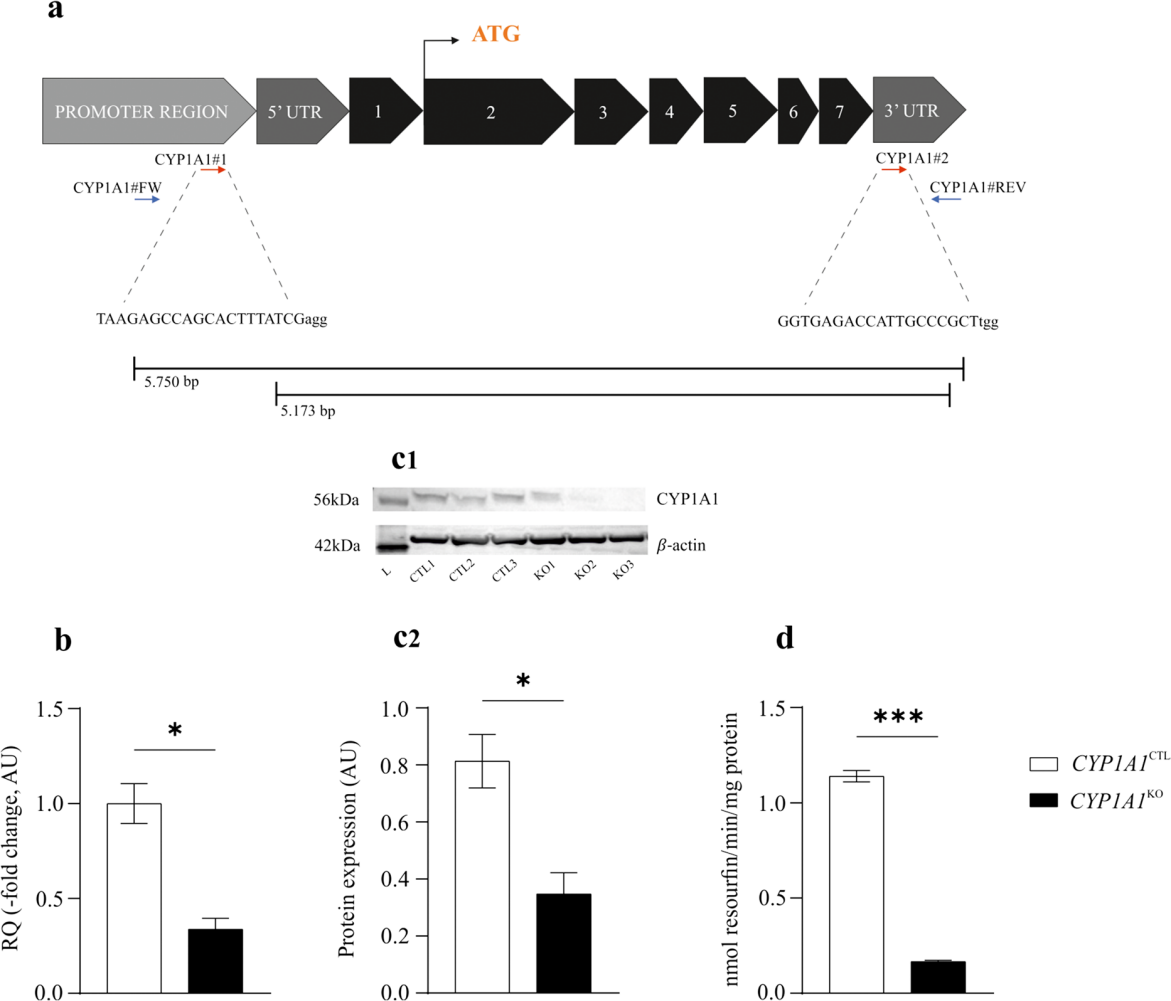
Description of CRISPR/Cas9 mediated *CYP1A1* KO and confirmatory assays. (**a**) The two gRNAs (red arrows) were designed to direct Cas9 machinery in *CYP1A1* promoter region and 3’ UTR. The Cas9 activity at these sites leads to a double-stranded break and a consequent deletion of ~ 5,173 bp. (**b**) Gene expression data (-fold change, arbitrary units, AU) are reported as the mean ± SEM of three biological replicates, each performed in duplicate. (**c1**) Cytochrome P450 1A1 immunoblotting, using β-actin as loading control. (**c2**) Densitometric analysis data are expressed in AU as the mean ± SEM of three biological replicates. (**d**) Cytochrome P450 1A1-dependent catalytic activity. The ethoxyresorufin *O*-deethylation (EROD) activity is expressed in nmoles min^−1^ mg^−1^ protein and represents the mean ± SEM of three biological replicates, each performed in duplicate. Statistical analysis: unpaired t-test with Welch’s correction. *: *p* < 0.05 and ***: *p* < 0.001, *CYP1A1*^KO^ vs. *CYP1A1*^CTL^ cells

To verify the correct deletion of the target gene, genomic DNA was extracted employing DNeasy Blood & Tissue Kit and quantified using the NanoDrop 1000 Spectrophotometer (Thermo Fisher Scientific, Waltham, MA, USA). Then, a PCR amplification was performed using 60 ng of DNA, Phire Hot Start II DNA Polymerase and primers flanking the deletion sites (Supplementary Table 2; Fig. 1a). The obtained PCR products (Supplementary Fig. 1b) were visualized by electrophoresis on 1.5% agarose gel, purified and sequenced in outsourcing (BMR Genomics, Padua, IT). To confirm the correct deletion of *CYP1A1*, the obtained sequences were analysed using MultAlin tool (http://multalin.toulouse.inra.fr/multalin/).

### RNA Extraction and qPCR

Cells (passages 23–26) were seeded in 6-well plates (50 × 10^3^ cells/well) and four days post-plating total RNA was isolated using the RNeasy Mini Kit (Qiagen) and quantified by Qubit RNA Assay Kit in a Qubit 2.0 Fluorometer (Life Technologies). The extracted RNA was used for both qPCR and RNA-seq analyses. As to qPCR, 1 µg total RNA was reverse-transcribed using the High Capacity cDNA Reverse Transcription Kit, following the manufacturer’s instructions. Then, the amplification was conducted using the LightCycler 480 Real-Time thermocycler (Roche Applied Science, Penzberg, Germany). The qPCR mix consisted of 1X SybrGreen PCR Master Mix, 300 nM forward and reverse primers (Supplementary Table 3) and 12.5 ng cDNA. The amplification plots were analysed using the LightCycler 480 SW 1.5. Finally, the relative quantification of the target gene was made using the ∆∆Ct method. For each experimental condition (i.e., *CYP1A1*^KO^ and *CYP1A1*^CTL^) three biological replicates, each performed in duplicate, were considered.

### Protein isolation and immunoblotting

Cells (passages 23–26) were seeded in Petri dishes (90 mm diameter) at a density of 3 × 10^5^ cells/Petri dish and four days post-plating protein isolation and quantification were performed as previously described (Iori et al. 2022). For immunoblotting assay, 20 µg of total proteins were loaded on the minigel and, then, transferred onto nitrocellulose filters as reported elsewhere (Zancanella et al. 2012). The membrane was incubated with rabbit anti-human CYP1A1 (1:1000 final concentration, overnight) and anti-ACTB (1:6000 final concentration, 2 h) primary antibodies, then with the horseradish peroxidase conjugated goat anti-rabbit (1:6000, 1.5 h) secondary antibody. The obtained immunopositive bands were then subjected to semi-quantification as reported elsewhere (Iori et al. 2022).

### Ethoxyresorufin O-deethylase assay

Cells (passages 23–26) were seeded in 6-well plates at a concentration of 50 × 10^3^ cells/well and were exposed to 1 nM PCB126 for 24 h at 37°C as previously reported (Pauletto et al. 2020) to enlarge the differences in terms of CYP1A1 catalytic activity between *CYP1A1*^CTL^ and engineered cells. Then, the culture medium was removed and cells were pre-treated with fresh medium without FBS and containing 30 nM (E)-2,3',4,5'-Tetramethoxystilbene (TMS) for 1 h at 37°C. Finally, cells were exposed to 5 µM ethoxyresorufin and 10 µM dicoumarol (a NAD(P)H:quinone oxidoreductase1 inhibitor) for 1 h at 37°C. At the end of the incubation time, the medium was collected and stored at -80 °C until LC–MS/MS analysis. The culture medium (300 µL) was spiked with 10 μL of the internal standard (IS, 300 ng/mL resorufin-d_6_) and then extracted with an equal volume (300 μL) of a mixture 1:1 hexane:dichloromethane (v/v). The water phase was re-extracted with methanol and the supernatant was dried under an air stream at 50 °C using a TurboVap evaporator (Zymarck, Hopkinton, MA, USA). Dried residues were dissolved in a reconstitution solution containing 0.1% formic acid in methanol (50%) and 0.1% formic acid in water (50%) and subjected to LC–MS/MS analysis. The chromatographic separation was achieved using an Accela 600 HPLC pump with a CTC automatic injector (Thermo Fischer Scientific, San Jose, CA, USA) equipped with a Hypersil Gold analytical column (50 × 3 mm, 5 μm; Thermo Fischer Scientific, San Jose, CA, USA). The method used a binary gradient with a mobile phase consisting of 0.1% formic acid (v/v) in H_2_O as solvent A and 0.1% formic acid (v/v) in methanol as solvent B. Optimized separation was obtained using the following gradient program (%A: %B): 75:25 at 0 min, 5:95 at 6 min, held at 5:95 until 8 min, 75:25 at 9 min, held at 75:25 until 11 min to re-equilibrate the system. The sample trays were maintained at 4^◦^C, the injection volume was 5 μL and the flow rate was set at 300 μL min^−1^. The mass detection was achieved by an LTQ XL ion trap (Thermo Fischer Scientific, San Jose, CA, USA) equipped with a heated electrospray ionisation probe. The mass spectra were acquired using positive-ion ESI mode. The two diagnostic fragments *m/z* 155 and 182 of resorufin were monitored. The X-calibur software (version 2.1) was used for system control, data acquisition and analysis. The CYP1A1 catalytic activity (nmoles min^−1^ mg^−1^ protein) was calculated by dividing the amount of resorufin metabolite detected in the medium by the incubation time and the total protein content. For each experimental condition (i.e., *CYP1A1*^KO^ and *CYP1A1*^CTL^) three biological replicates, each performed in duplicate, were used.

### RNA-sequencing analysis

Libraries construction (QuantSeq 3' mRNA-Seq Library Prep Kit FWD, Lexogen GmbH, Austria) and sequencing (Illumina Novaseq 6000, single-end 75 bp) were performed at the NGS facility of the University of Padova. For each experimental condition (*CYP1A1*^KO^ and *CYP1A1*^CTL^) three biological replicates were considered. Obtained raw reads were trimmed using the BBDuk program (BBTools suite) and then mapped to *Bos taurus* ARS-UCD1.2 reference genome as previously reported (Pauletto et al. 2020). Pair-wise comparisons between *CYP1A1*^KO^ and *CYP1A1*^CTL^ samples were carried out to highlight transcriptional changes induced by *CYP1A1* KO, setting a maximum False Discovery Rate (FDR) of 0.05. The ClusterProfiler package (Yu et al. 2012) was then implemented in R environment to functionally interpret significant differentially expressed genes (DEGs) through Gene Ontology (GO) and Kyoto Encyclopedia of Genes and Genomes (KEGG) over-representation tests. The mRNA expression of the genes of interest was reported as logarithm of counts per million (logCPM), with a* p*-value threshold set at 0.05.

### Statistical analysis

Statistical analysis was conducted using GraphPad Prism software (version 9.5.1, San Diego, CA, USA). Unpaired t-test with Welch’s correction was used. A value of *p* < 0.05 was considered significant.

## Results

### Knockout of *CYP1A1* in BFH12 cells

The RNP-complex approach was applied to obtain *CYP1A1* KO in BFH12 cells. A schematic representation of the Cas9-gRNA complexes targeting the deletion sites is reported in Fig. 1. Transfection efficiency was ~ 90% (Supplementary Fig. 1a). Clonal expansion, PCR amplification and Sanger sequencing of the targeted genomic region were executed, allowing the selection of a *CYP1A1*^KO^ clone used in the following steps.

### Cytochrome P450 1A1 expression and catalytic activity in *CYP1A1*^KO^ and *CYP1A1*^CTL^ cells

The CYP1A1 mRNA levels and the corresponding coded protein amount were measured by specific qPCR and immunoblotting assays, respectively. Engineered cells (*CYP1A1*^KO^) showed reduced *CYP1A1* mRNA (~ 70%) and apoprotein (~ 80%) amounts if compared to *CYP1A1*^CTL^ cells (Fig. 1b, c). A similar result was obtained at the catalytic activity level, since EROD activity was significantly decreased (~ 90%) in *CYP1A1*^KO^ cells (Fig. 1d).

### Analysis of the differentially expressed genes

**Fig. 2:**
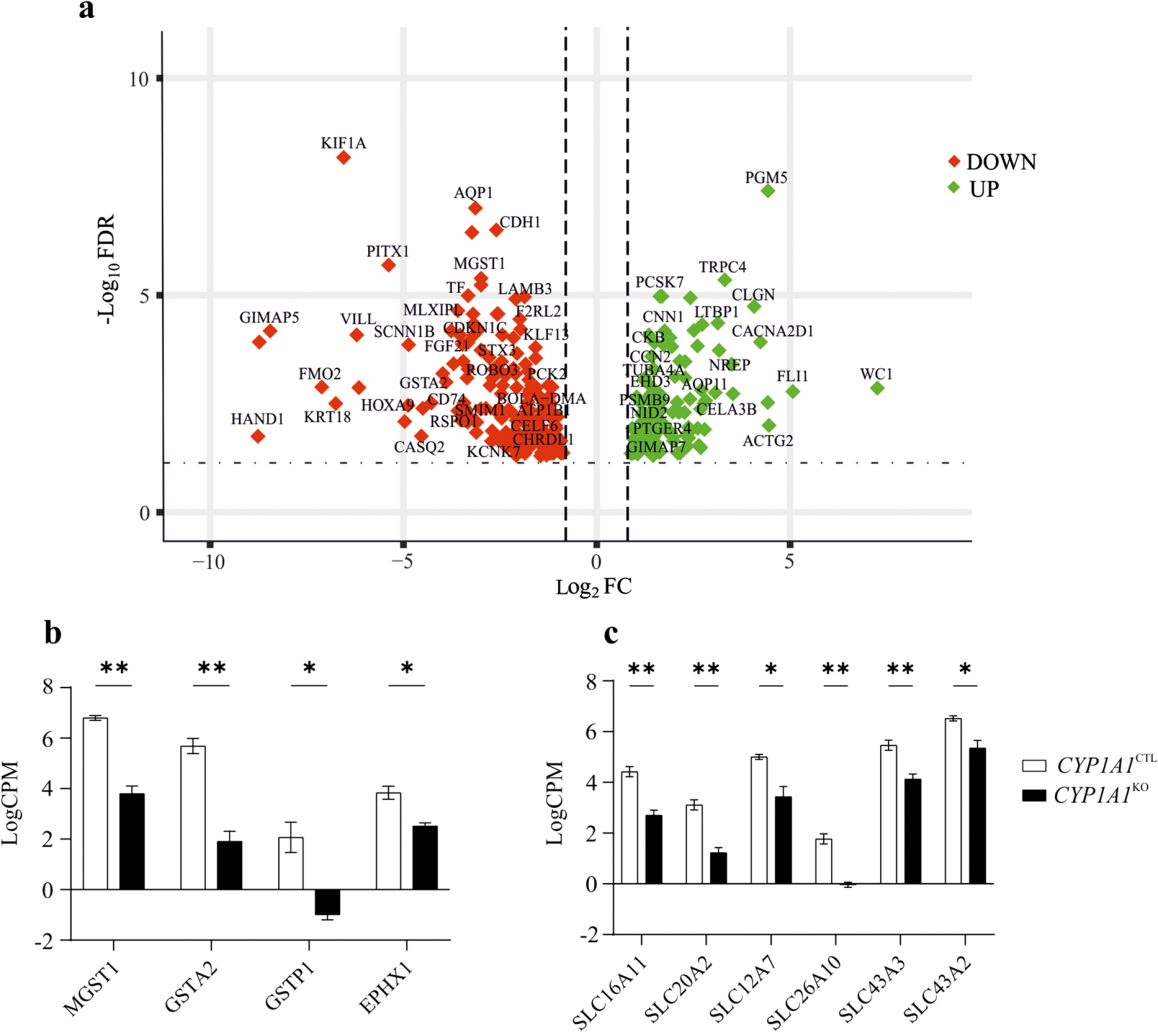
(**a**) Volcano plots representing the differentially expressed genes (DEGs) identified by comparing *CYP1A1*^KO^ and *CYP1A1*^CTL^ cells, using a FDR threshold of 0.05. (**b, c**) Focus on the gene expression of glutathione transferases (*GSTs*), epoxide hydrolase 1 (*EPHX1*) and members of the solute carrier (SLC) family of drug transporters. Data are expressed as the mean LogCPM ± SEM of three biological replicates

A total of 27,556,698 raw reads were obtained and deposited in GeneBank under the BioProject accession number ID PRJNA1048728. The Multidimensional Scaling (MDS) plot provided an unsupervised clustering of samples. The first dimension clearly separated each experimental condition (i.e., *CYP1A1*^KO^ and *CYP1A1*^CTL^). A pair-wise comparison aimed at visualizing transcriptional changes induced in *CYP1A1*^KO^ cells identified 344 DEGs (141 and 203 up- and down-regulated genes, respectively; Fig. 2a; Supplementary Table 4). When analysing the list of DEGs, an impact of *CYP1A1* deletion on XMEs was observed; in particular, three glutathione *S*-transferase (GST) isoforms (i.e., *GSTA2*, *GSTP1* and *MGST1*) and the epoxide hydrolase 1 (*EPHX1*) were down-regulated in *CYP1A1*^KO^ compared to *CYP1A1*^CTL^ cells (Fig. 2b). A similar behaviour was observed for several members of the solute carrier (SLC) family of drug transporters, that overall showed a reduction in their mRNA levels in *CYP1A1*^KO^ cells (Fig. 2c).

### Functional analysis of differentially expressed genes

**Fig. 3:**
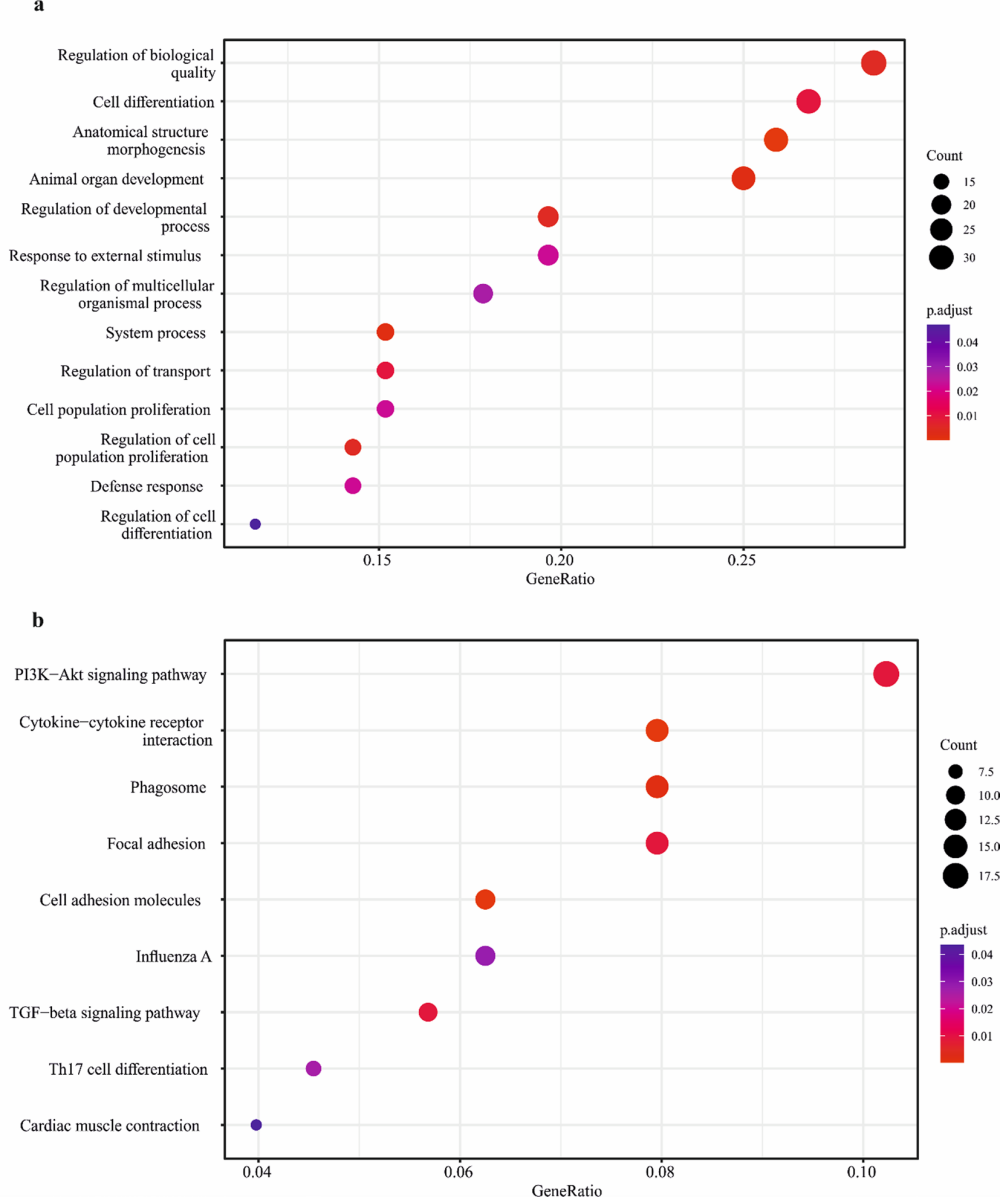
GO (**a**) and KEGG (**b**) enrichment analysis of DEGs in *CYP1A1*^KO^ vs *CYP1A1* cells. Gene ratio is the percentage of DEGs over the total number of genes in each pathway. Count (dot size) represents the number of DEGs enriched in a certain pathway. The colour gradient represents the adjusted significance level (p. adjusts), according to the Benjamin-Hochberg method

The functional analysis revealed 13 enriched Biological Processes (BPs) and 9 enriched KEGG pathways (Fig. 3a, b; Supplementary Table 5a, b).

Concerning the enriched BPs, terms like ‘System process’, ‘Regulation of biological quality’, ‘Regulation of transport’, ‘Defence response’, ‘Response to external stimulus’ and ‘Regulation of multicellular organismal processes’ clearly suggest an alteration of biological and metabolic processes in *CYP1A1*^KO^ cells. These terms were represented by a number of genes involved in lipid metabolism, such as the Fatty Acid Binding Protein 5 (*FABP5*) and the Peroxisome Proliferator Activated Receptor Gamma (*PPARG*). Other genes affected by *CYP1A1* deletion and belonging to the aforementioned BPs are Chemerin 1 (*CHN1*) and Annexin-A6 (*ANXA6*); the former is associated with metabolic and immune dysregulation, while the second one encodes for a Ca2 + -dependent membrane binding protein. Moreover, the Prostaglandin-Endoperoxide Synthase 2 (*PTGS2*), a key enzyme in prostaglandin H_2_ biosynthesis, was up-regulated in *CYP1A1*^KO^ cells. Conversely, the Dehydrogenase/Reductase 9 (*DHRS9*), thought to be involved in the retinol metabolism, decreased its expression in genetic modified cells. Additionally, a member of the aldo–keto reductase (AKR) Superfamily (i.e., *AKR1B1*), which catalyses the reduction of carbonyl groups to primary and secondary alcohols using electrons from NADPH, was shown to be inhibited in *CYP1A1*^KO^ cells.

An impact on cell cycle regulation and proliferation was also indicated, as demonstrated by the enrichment of ‘Anatomical structure morphogenesis’, ‘Animal organ development’, ‘Regulation of cell population proliferation’, ‘Regulation of developmental process’, ‘Cell differentiation’, ‘Cell population differentiation’ and ‘Regulation of cell differentiation’ BP terms. Among them, we found several up-regulated genes encoding for actin proteins, such as the Actin Alpha 1 (*ACTN1*), the Actin Alpha 2 Smooth Muscle (*ACTA2*) and the Actin Gamma 2 Smooth Muscle (*ACTG2*), along with the inhibin subunit beta A (*INHBA*). The up-regulation of the Cyclin D2 (*CCND2*) and the ETS Variant Transcription Factor 1 *(ETV1*), whose dysregulation may trigger initiation and progression of multiple types of tumours (Ding et al. 2019; Eid and Abdel-Rehim 2019) was also highlighted. On the other hand*,* Troponin C (*TNNC1*), a member of the troponin complex which may exert tumour suppression activities (Kim et al. 2020), was found to be down-regulated in the engineered cells. The Angiopoietin 2 (*ANG2*), having a role in hepatocyte proliferation (Morse et al. 2019), and the ST14 Transmembrane Serine Protease Matriptase (*ST14*), whose inhibition was associated with tumour cell invasion and metastasis (Yan and Yan 2015), were also down-regulated.

KEGG enriched analysis pointed out the over-representation of pathways linked to cell cycle and biological processes regulation (i.e., ‘TGF-beta signalling pathway’, Cell adhesion molecules’, ‘Focal adhesion’, ‘Cardiac muscle contraction’, and ‘PI3K-Akt signalling pathway’) as previously highlighted by GO analysis. Specifically***,*** both Versican (*VCAN*) and Phosphoinositide-3-kinase regulatory subunit 3 (*PIK3R3*) genes, playing modulatory roles in cell proliferation and differentiation (Guo and Liu 2021), were up-regulated upon *CYP1A1* deletion. Likewise, some members of integrin family of transmembrane receptors (i.e., *ITGA1*, *ITGA5* and *ITGB3* isoforms) showed increasing mRNA levels following the *CYP1A1* KO. On the other end, cadherin *1* (*CDH1*) as well as several other genes contributing to iron and lipid homeostasis, i.e., transferrin (*TF*), the inhibitor of DNA binding 1, the HLH Protein (*ID1*), the Bone Morphogenetic Protein 4 (*BMP4*), and the TGFB Induced Factor Homeobox 1 (*TGIF1*), were down-regulated in genetically modified cells. Also, the Insulin Like Growth Factor 2 (*IGF2*), along with the gene encoding for the mitochondrial Phosphoenolpyruvate Carboxykinase 2 (*PCK2*), an enzyme participating in gluconeogenesis pathway, resulted inhibited in *CYP1A1*^KO^ cells.

The enrichment of pathways strictly related to inflammatory processes and immunity, e.g., ‘Cytokine-cytokine receptor interaction’, ‘Phagosome’ and ‘Influenza A’ was also observed. In particular, a number of interleukins (ILs) such as *IL6* and *IL18,* as well as the C-X-C motif chemokine ligand 9 and 10 (*CXCL9* and *CXCL10*, respectively) were up-regulated. Conversely, several members of the bovine Major histocompatibility complex II (MHC II) like *BOLA*-*DRA*/-*DMA*/-*DMB*, the Complement C1r (*C1R*) and the anti-inflammatory cytokine IL1 Receptor Antagonist (*IL1RN*) showed an opposite behaviour (i.e., down-regulation) in engineered cells.

## Discussion

In human and laboratory species, the *CYP1A* subfamily takes part in the oxidative metabolism of a variety of exogenous and endogenous compounds. To ascertain the specific role of *CYP1A* in xenobiotic and drug metabolism, *CYP1A* KO rodent models have been developed (Kapelyukh et al. 2019; Lu et al. 2023). Cattle is a worldwide relevant food-producing species, potentially exposed to xenobiotics either intentionally (e.g., drugs) than not (e.g., food and feed contaminants and environmental pollutants). Upon the uptake from the site of absorption, these xenobiotics reach the liver and undergo detoxification or bioactivation reactions catalysed by XMEs. The obtained derivatives (or the parental compound itself) may give residues in edible tissue and dairy food products, thus resulting potentially harmful for consumers. Few studies have proved the involvement of bovine *CYP1A* isoform in the hepatic biotransformation of xenobiotics, and also its induction by prototypical inducers or some environmental pollutants (Maté et al. 2019; Pauletto et al. 2020; Giantin et al. 2018). Hence, to better decipher the molecular mechanisms involved in *CYP1A* regulation and its role in cattle xenobiotic metabolism, we applied the CRISPR/Cas9 technology in BFH12 cells to delete *CYP1A1* gene, the prevalent *CYP1A* isoform in this bovine cell line (Giantin, personal data).

Once we obtained the *CYP1A1*^KO^ clone, whose correct deletion was confirmed by DNA Sanger Sequencing, we run confirmatory studies at the transcriptional and protein level. Results of mRNA and coded protein expression were in accordance, thereby confirming the almost complete *CYP1A1* ablation. It should be mentioned that a residual expression was noticed at the gene and protein level (~ 30% and ~ 20%, respectively). This result was not surprising, since a potential contamination by non-homozygous KO cells, together with naïve cells, is a phenomenon that can occur when CRISPR/Cas9 system is applied in vitro (Heintze et al. 2021). As to the CYP1A1-dependent enzyme activity, we measured EROD. Worth to mention, the pre-incubation with TMS and the contemporary addition of dicumarol and ethoxyresorufin allowed us to assess the CYP1A1 activity without the interference of CYP1B1 and DT-diaphorase (Iba et al. 2010). A statistically significant reduction (up to 90%) of EROD activity was recorded in *CYP1A1*^KO^ cells; the small residual enzyme activity in genetic modified cells may be again ascribed to the presence of heterozygous and naïve cells, as previously hypothesised for mRNA and protein expression levels.

The challenge of establishing a reliable genetically modified cell model that ensures reproducible results is a matter of concern in studies relying on genetically modified cell lines. As described above, in the present investigation an incomplete silencing of *CYP1A1* was observed. To ameliorate such a result, the sorting of transfected cells thanks to the fluorescent marker might be an option. However, the delivery of the RNP complex into cells does not guarantee a successful cleavage by the Cas9 enzyme, so an inhomogeneous cell population would be still expected. Considering the aforementioned challenge, the cell passage number is considered a critical factor contributing to the genotypic variability of *CYP1A1*^KO^ cells during time. Nevertheless, this limitation could be mitigated by narrowing the number of cell passages; this approach was applied in the present study, with the aim of preventing the dominance of naïve cells over engineered ones and ensuring the reproducibility of the experimental results.

Whenever using a gene editing approach like CRISPR/Cas9, it is important to ascertain whether gene KO will have remarkable or insignificant effects on the global transcriptome. To this purpose, we performed a comparative RNA-seq analysis to identify the possible transcriptional perturbations induced by *CYP1A1* KO. Noteworthy, none of the observed DEGs coincided with the off-target genes predicted for the gRNAs used; thus, their dysregulation is attributable only to the *CYP1A1* KO.

Interestingly, *CYP1A1* deletion highlighted a direct effect on the mRNA expression of some phase II XMEs. Specifically, *GSTA2*, *GSTP1* and *MGST1*, members of the GST class of conjugative XMEs known to be involved in the cellular defence against toxic and carcinogenic compounds (Potęga 2022), were inhibited as a consequence of *CYP1A1* KO. The same consideration can be drawn for *EPHX1*, another critical gene responsible for the conversion of an epoxide to dihydrodiol which, in turn, may be further conjugated and excreted from the body (Gautheron and Jéru 2021). Such a result might be considered a limitation of the *CYP1A1*^KO^ cell model if the reduced mRNA expression is associated to a defective mechanism of chemical biotransformation and detoxification. Nevertheless, it might be emphasised that *GSTA2, MGST1 and EPHX1* mRNA expression still persisted at good levels in the engineered cell line (logCPM > 2); thus, a detoxifying activity is anyway expected. Conversely, the evidence of a potential interaction at the regulatory level between *CYP1A1* and the abovementioned phase II XMEs might be considered of value and worth of further investigations.

It’s known that SLC transporters are a class of proteins involved in transport of ions, nutrients, and xenobiotics across biological membranes. In this study, a number of SLC family members were down-regulated upon *CYP1A1* deletion, including those engaged in hepatic lipid and phosphate homeostasis (i.e., *SLC16A11* and *SLC20A2,* respectively) along with *SLC12A7*, an afflux transporter participating in cell volume regulation (Zhang et al. 2023). These results suggest a strong impact of *CYP1A1* deletion on cell metabolic activity and volume homeostasis. The alteration of metabolic processes and cell steadiness in *CYP1A1*^KO^ cells is corroborated by DEGs functional analysis, and particularly the enrichment of BPs terms related to the regulation of biological and metabolic cellular processes (e.g., ‘System process’, ‘Regulation of biological quality’, ‘Regulation of multicellular organismal processes’).

In *CYP1A1*^KO^ cells, a down-regulation of *TF*, a gene encoding for a glycoprotein responsible for the transport of iron to all proliferating cells in the body, was highlighted (Kawabata 2019). This might result in alterations of iron homeostasis. In this respect, further evidence is the down-regulation of certain BMP subfamily members (i.e., *BMP4*) together with *ID1*, a major downstream transcriptional target of BMP signalling; the latter one is known to play a role in the regulation of iron homeostasis, as demonstrated in previous hepatic in vitro studies (Charlebois and Pantopoulos 2021).

Also, the metabolism of retinoids was somehow modulated by *CYP1A1* deletion, as shown by *DHRS9* and *AKR1B1* genes down-regulation. The former gene is a NAD(P)(H)-dependent oxidoreductase catalysing the interconversion of retinol and retinal, while the second one exhibits retinaldehyde reductase activity.

Noteworthy, in this study *IGF2* mRNA level was strongly decreased in *CYP1A1*^KO^ cells. This would suggest an impairment of mitochondrial homeostasis. Indeed, the in vitro hepatic *IGF2* knockdown resulted in reduced mitochondrial membrane potential and increased production of reactive oxygen species (Gui et al. 2021). In addition, the mitochondrial *PCK2* gene, which converts oxaloacetate to phosphoenolpyruvate, was down-regulated, hence corroborating our hypothesis. A dysregulation of mitochondrial functions is directly linked to the imbalance of cellular metabolic processes, and this can favour the onset of the Non-Alcoholic Fatty Liver Disease (NAFLD), characterized by an excessive lipid accumulation in the liver (Zheng et al. 2023). In our study, we observed an up-regulation of genes associated with a dysregulation of lipid homeostasis such as *PPARγ*, whose involvement in hepatic fat accumulation, followed by NAFLD, has been previously demonstrated in mouse models (Wang et al. 2020). Intriguingly, *PTGS2* mRNA level was strongly increased in *CYP1A1*^KO^ cells. In the bovine species such an increase has been previously reported in liver slices where the addition of polyunsaturated fatty acids was shown to cause oxidative stress and lipid peroxidation (Fortin et al. 2017). On a comparative basis, this gene shows some functional similarities between bovine and rat species, as in the latter it plays an important role in NAFLD development through the dysregulation of lipid metabolism (Hsieh et al. 2009). Furthermore, the *FABP5* gene, encoding for a lipid-binding protein participating in the intracellular transports of fatty acids (Xu et al. 2022), was up-regulated; this might possibly increase the uptake of fatty acids, which in turn may lead to hepatic lipid accumulation. The disruption of lipid homeostasis was also corroborated by the down-regulation of *TGIF1*, as higher hepatic lipid accumulation was observed in *TGIF1*^KO^ mice (Pramfalk et al. 2014).

In the present study, an over-representation of BPs terms (e.g., Anatomical structure morphogenesis’, ‘Animal organ development’, ‘Regulation of cell population proliferation’) and KEGG pathways (e.g., ‘TGF-beta signaling pathway’, Cell adhesion molecules’, ‘and ‘PI3K-Akt signaling pathway’) related to cell cycle regulation, growth and proliferation, and mostly linked to carcinogenesis, were observed. As an example, the *PIK3R3* gene was up-regulated in *CYP1A1*^KO^ cells. This transcript is a member of the PI3K/Akt signalling pathway, whose abnormal activation may drive to malignant outcomes. Specifically, the *PIK3R3* gene is up-regulated in a number of tumours, including HCC, and exerts oncogenic functions (Lin et al. 2023). Another example are inhibin proteins. These ones are members of the TGFβ superfamily which controls, among the others, cell proliferation and differentiation pathways in several organs, including the liver. Among them, the *INHBA* gene plays a role in the occurrence and progression of several types of cancer, such as adenocarcinoma and breast cancer (Li et al. 2020; Yu et al. 2021); in *CYP1A1*^KO^ cells the *INHBA* gene was actually up-regulated. Furthermore, the *VCAN* gene, encoding for a proteoglycan involved in cell adhesion, proliferation and migration, was up-regulated, too. Intriguingly, this transcript has been previously associated with cancer progression, thanks to *INHBA* up-regulation (Guo and Liu 2021). Conversely, the *TNNC1* gene (a troponin complex member) was down-regulated. Although some troponin complex members are overexpressed in several cancer types, a recent study clearly proved the tumour suppressor activity of *TNNC1* (Kim et al. 2020). Likewise, the *HMBG3* gene, which is overexpressed during hepatocytes malignant transformation (Zheng et al. 2018), was down-regulated in genetically modified cells. We claim that *HMGB3* down-regulation might represent a cells attempt to escape from becoming cancerous; notably, this is supported by a recent study in which *HMGB3* down-regulation may trigger apoptosis (Sharma et al. 2022). As a whole, based on these evidences, we might consider this in vitro* CYP1A1*^KO^ model useful for adverse outcome pathways and carcinogenesis research. Indeed, CYP1A1 is linked to important physiological and cell growth regulatory processes, through the involvement of AhR, other than the pathways induced by the metabolic activation of chemical compounds (Androutsopoulos et al. 2009). The AhR is in fact involved in various cell signalling pathways critical to cell cycle regulation and normal homeostasis, and the dysregulation of these pathways is known to be implicated in tumor progression (Androutsopoulos et al. 2009). Since AhR and CYP1A1 are subjected to an autoregulatory feedback loop through an endogenous ligand (Chiaro et al. 2007), this mechanism of autoregulation could be at the basis of the AhR-dependent induction of cell cycle regulation, cell growth and proliferation, and carcinogenesis pathways observed in the BFH12 *CYP1A1*^KO^ cell model in absence of exogenous AhR ligands.

Tumour metastasis requires the cancer cells to break off from original site and spread to other parts of the body. In such a process, a major role is played by the epithelial-mesenchymal transition (EMT), which involves a dynamic remodelling of cytoskeleton actin filaments. Indeed, actin filaments contribute to metastasis progression, whereas actin proteins protect tumour cells during their transport in the bloodstream, thus favouring their attachment to the new tumour site (Izdebska et al. 2018). Another important process of EMT is the down-regulation of the epithelial marker E-cadherin (Bertran et al. 2013). In our study, several genes encoding for actin proteins (i.e., *ACTN1*, *ACTA2* and *ACTG2*) were up-regulated in *CYP1A1*^KO^ cells, while *CDH1* showed an opposite behaviour, hence leading us to speculate about a predisposition of *CYP1A1*^KO^ cells to EMT.

Last but not least, inflammation and immune response processes were somehow dysregulated in *CYP1A1*^KO^ cells. This is highlighted by the enrichment of KEGG pathways like ‘Cytokine-cytokine receptor interaction’, ‘Phagosome’, and ‘Influenza A’. Some members of the bovine MHC II (e.g., *BOLA*-*DRA*/-*DMA*/-*DMB*) were down-regulated in *CYP1A1*^KO^ cells. The down-regulation of MHC genes is a well-known phenomenon occurring in tumour microenvironment (Gonzalez et al. 2018). However, two chemokine ligands, i.e. *CXCL9* and *CXCL10*, were up-regulated, and such an up-regulation was previously associated with liver injury, e.g., cirrhosis and HCC (Yu et al. 2022).

In conclusion, in this study we successfully applied the CRISPR/Cas9 technology to build up a new bovine *CYP1A1*^KO^ in vitro model starting from BFH12 cells. Confirmatory assays conducted in *CYP1A1*^KO^ cells proved the almost complete loss of *CYP1A1* gene, mRNA, coded protein, and catalytic activity. Overall, the bovine in vitro model we developed in this study represents a valuable resource to investigate bovine liver *CYP1A1* gene regulation and CYP1A1-mediated xenobiotic metabolism, which are of extreme importance for ensuring human health in a one-health perspective. Indeed, CYPs participate to the bioactivation process of a number of carcinogens and genotoxic compounds that can be potentially found in cattle-derived food products for human consumption. Intriguingly, we recently applied this modified cell line to further investigate the specific role of CYP1A1 in the mechanistic toxicology of AFB1, a well-known hepatotoxic and carcinogenic food and feed contaminant (Iori et al. submitted). Thus, in perspective this cell model could be useful in the field of safety assessment to screen the potential harmful effects of chemicals, prior to their use in the cattle industry. Nevertheless, a deep perturbation of the BFH12 cell transcriptome as a result of *CYP1A1* KO has been shown, particularly an impact on cell cycle regulation, proliferation and detoxification processes, as well as on iron, lipid and mitochondria homeostasis. Consequently, *CYP1A1*^KO^ cells may be also useful for improving the knowledge on the role played by bovine *CYP1A1* in cell homeostasis.

## Supplementary Information

Below is the link to the electronic supplementary material.

Supplementary file1 (DOCX 181 KB)

Supplementary file2 (XLSX 48 KB)

Supplementary file3 (XLSX 16 KB)

## Data Availability

Raw Illumina Sequencing Data have been deposited in GenBank (SRA) under the BioProject ID PRJNA1048728.
