# Supplementary material for: Establishment and characterization of cytochrome P450 1A1 CRISPR/Cas9 Knockout Bovine Foetal Hepatocyte Cell Line (BFH12)
Source: Cell Biol Toxicol. 2024 Mar 26;40(1):18. doi: 10.1007/s10565-024-09856-7 (PMC10963470; doi:10.1007/s10565-024-09856-7)
Supplement: Supplementary file 1 — Supplementary file1 (DOCX 181 KB) [file 10565_2024_9856_MOESM1_ESM.docx]

**Establishment and Characterization of Cytochrome P450 1A1 CRISPR/Cas9 Knockout Bovine Foetal Hepatocyte Cell Line (BFH12)**

Silvia Iori^a^, Caterina D’Onofrio^a^, Nihay Laham-Karam^b^, [Isidore Mushimiyimana](https://pubmed.ncbi.nlm.nih.gov/?term=Mushimiyimana+I&cauthor_id=34320216)^b^, Lorena Lucatello^a^, Rosa Maria Lopparelli^a^, Maria Elena Gelain^a^, Francesca Capolongo^a^, Marianna Pauletto^a^, Mauro Dacasto^a^ & Mery Giantin^a,*^

*^a^ Department of Comparative Biomedicine and Food Science, University of Padua, Viale dell’Università 16, Legnaro, 35020 Padua, Italy*

*^b^ University of Eastern Finland, A.I. Virtanen Institute for Molecular Sciences, Neulaniementie 2, 70211 Kuopio, Finland*

*^c^ Department of Animal Medicine, Production and Health, University of Padua, Viale dell’Università 16, Legnaro, 35020 Padua, Italy*

* corresponding author at: Department of Comparative Biomedicine and Food Science, University of Padua, Viale dell’Università 16, Legnaro, 35020 Padua, Italy. E-mail address: mery.giantin@unipd.it (M. Giantin).

**Supplementary Materials**

**Table 1***.* **Scores of the gRNAs designed for CYP1A1 KO**. MIT and CFD specificity scores predict the likelihood of off-target cleavage elsewhere in the genome by the selected gRNA; it is graded on a scale from 0 to 100, with 100 indicating the highest accuracy. The off-target count represents the number of possible off-targets in the genome; in detail, the individuated off-targets were primarily associated with intronic and intergenic regions, and were characterized by ≥ 2 mismatches. The Doench score is a forecast of the efficacy in cutting the target sequence using the chosen gRNA; it spans a scale from 0 to 100, with 100 denoting the highest level of effectiveness.

| **Guide ID** | **MIT Specificity Score** | **CFD Specificity Score** | **Off Target Count** | **Doench Score** |
| --- | --- | --- | --- | --- |
| CYP1A1#1 | 91 | 95 | 57 | 64 |
| CYP1A1#2 | 93 | 96 | 32 | 42 |

**Table 2***.* List of primers used for genotyping analysis.

| **Primers ID** | **Sequence (5’ 🡪 3’)** |
| --- | --- |
| CYP1A1#FW | AGTGTTCAGTGCGTGCAAAAAT |
| CYP1A1#REV | TGACCATGAAGTACGCCCGC |

**Table 3.** List of primers used for qPCR analysis.

| **Target gene** | **Primer** | **Primer sequence 5’ 🡪 3’** |
| --- | --- | --- |
| *CYP1A1* | Forward | GACCTGAATCAGAGGTTCTACGTCT |
|  | Reverse | CCGGATGTGACCCTTCTCAA |
| *RPLP0** | Forward | CAACCCTGAAGTGCTTGACAT |
|  | Reverse | AGGCAGATGGATCAGCCA |

* Ribosomal protein, large, P0 (RPLP0) was used as reference gene. From: T.L. Robinson, I.A. Sutherland, J. Sutherland, Validation of candidate bovine reference genes for use with real-time PCR, Vet Immunol Immunopathol 115 (2007) 160–165. https://doi.org/10.1016/j.vetimm.2006.09.012.

*
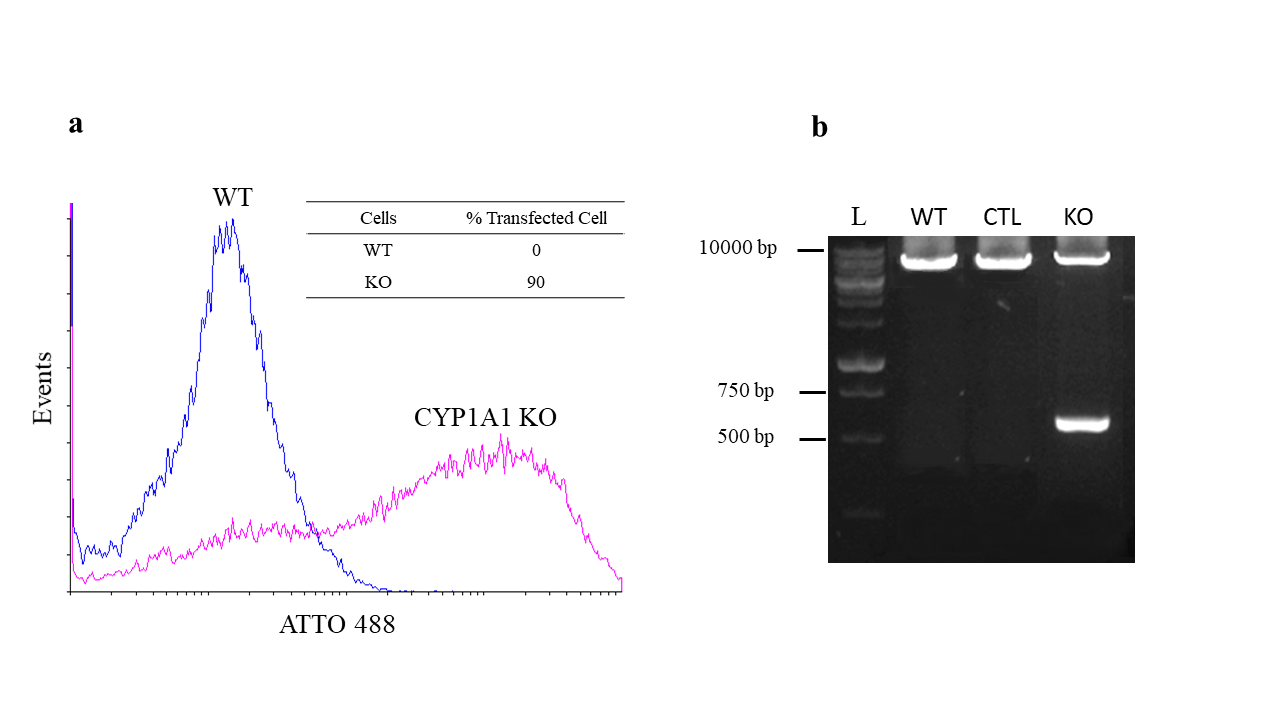
*

**Figure 1.** (**a**) Flow cytometry analysis of KO (violet) and native wild-type (WT, blue) cells. (**b**) Agarose gel electrophoresis to confirm the correct deletion. The PCR fragment length corresponded to ∼ 5.750 for WT and CTL cells, and ∼ 577 bp for KO cells.
